# Rolando Fracture Complication After Nonstandard Surgical Treatment

**DOI:** 10.7759/cureus.91666

**Published:** 2025-09-05

**Authors:** Jakub Florek, Filip Georgiew, Pawel Florek, Oles Petrovych, Sebastian Janowiec

**Affiliations:** 1 Department of Orthopaedics and Traumatology, Rydygier Hospital, Brzesko, POL; 2 Faculty of Health Science, University of Applied Science, Tarnów, POL

**Keywords:** arthroplasty, carpometacarpal, cmc, complications, dislocation, endoprosthesis, rolando fracture, thumb

## Abstract

The article describes the case of a patient who previously suffered a Rolando fracture and was treated in an innovative way using a dual mobility endoprosthesis of the left carpometacarpal (CMC) joint of the thumb. Three years after the surgery, as a result of an injury (falling down the stairs), the endoprosthesis dislocated. Dislocation of the thumb CMC joint prosthesis did not affect the clinical condition of the hand. From the assessed parameters, only a slight decrease in the global grip strength of the hand was observed. The patient himself assessed his level of fitness as satisfactory. We decided to try conservative treatment of the patient. The use of conservative treatment and observation allowed for the avoidance of a complicated revision surgery.

## Introduction

A Rolando fracture is a rare intra-articular Y- or T-shaped fracture of the base of the first metacarpal bone. Conservative treatment is usually ineffective. Surgical treatment aims to achieve anatomical reduction, restore the fractured bones to their original position, and achieve stabilization of the fragments. In the next stage, early physiotherapy is initiated to maintain the range of motion, improve the functional status, and reduce the intensity of pain. Treatment of a Rolando fracture is difficult due to its inherent instability and comminuted patterns. For this reason, complications may occur during treatment: loss of reduction, joint incongruity, and the potential development of osteoarthritis [1].

We recently reported an interesting case of a patient after an injury to the base of the first phalanx. The patient was diagnosed with a Rolando fracture. To enable the patient to quickly return to full functional capacity of the upper limb while minimizing the risk of degenerative changes (osteoarthritis and disability) in the joint, an innovative technique was used, implanting a dual mobility endoprosthesis into the carpometacarpal (CMC) joint of the thumb. In the 24-month follow-up period, both clinical and radiological treatment results were very good [2]. Unfortunately, after this period, the patient suffered another hand injury, which occurred as a result of a fall down the stairs. Its consequence was the dislocation of the implanted endoprosthesis. In this article, we presented the current assessment of the patient's clinical and radiological condition.

## Case presentation

**Figure 1:**
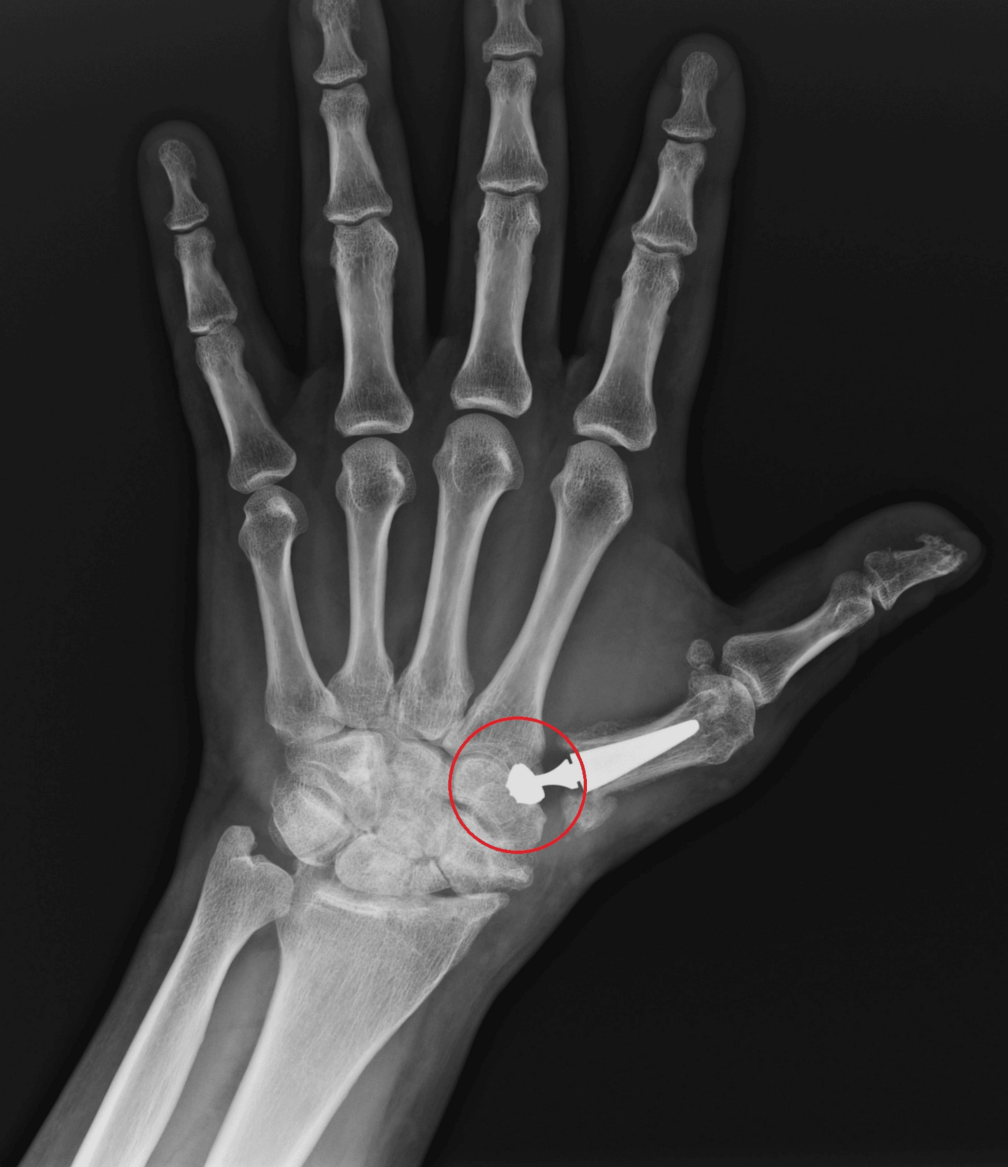
X-ray in the A-P projection obtained two years after surgery The correct positioning of all endoprosthesis elements (head of a double mobile CMC thumb prosthesis and metal acetabular shell) is marked in red. A-P: anterior-to-posterior

**Figure 2:**
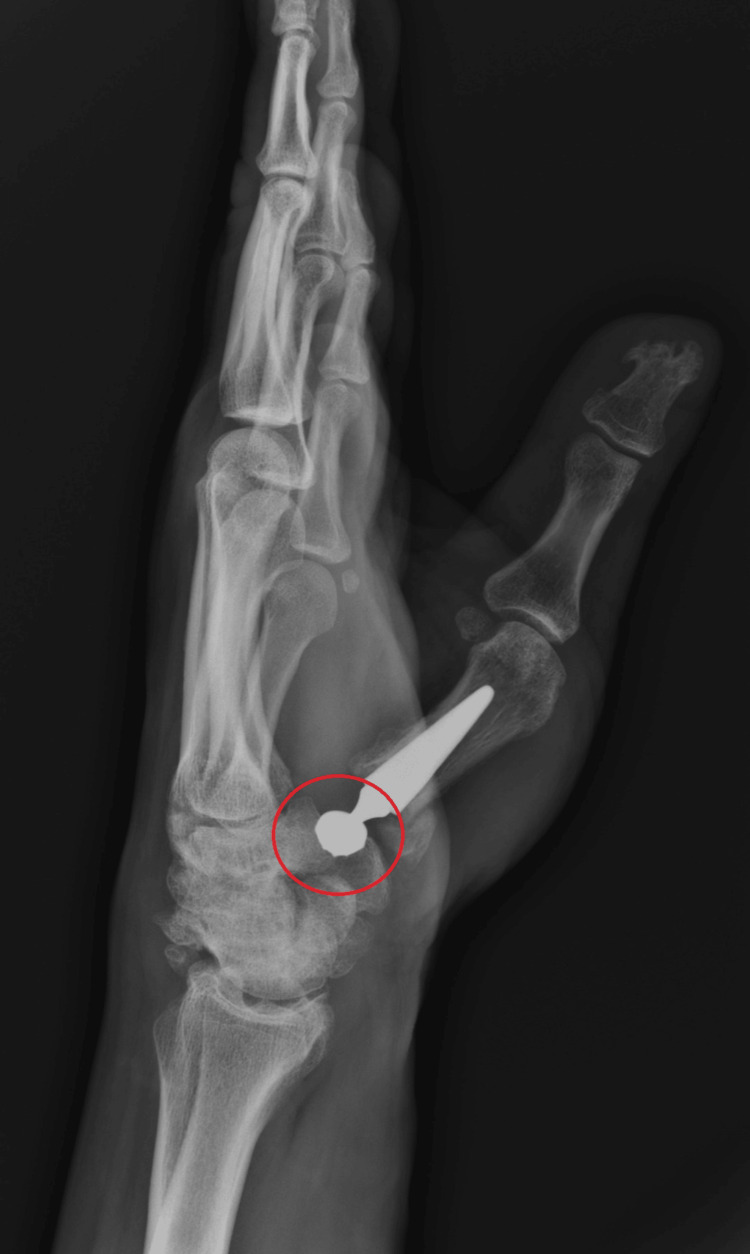
X-ray in the lateral projection obtained two years after surgery The correct positioning of all endoprosthesis elements (head of a double mobile CMC thumb prosthesis and metal acetabular shell) is marked in red. CMC: carpometacarpal

The article describes the case of a patient who previously suffered a Rolando fracture and was treated in an innovative way using a dual mobility endoprosthesis of the left CMC joint of the thumb. Control X-rays two years after implantation show the correct positioning of all endoprosthesis elements (Figures 1, 2).

**Figure 3:**
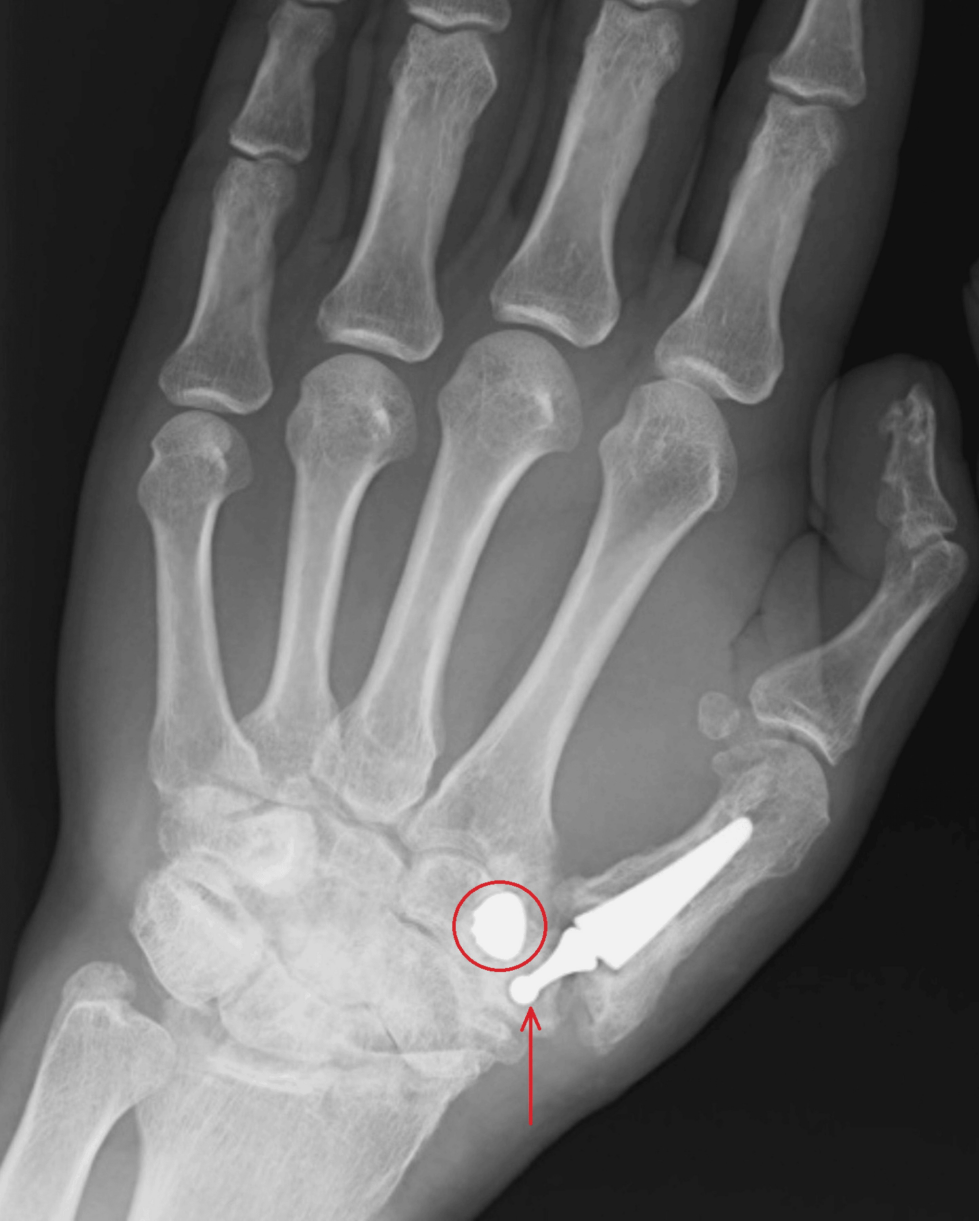
X-ray in A-P projection obtained three years after surgery The figure shows the dislocation of the implanted endoprosthesis elements. The red arrowhead marks the dislocated head of the double mobile CMC thumb prosthesis. The open circle marks the metal acetabular shell. A-P: anterior-to-posterior; CMC: carpometacarpal

**Figure 4:**
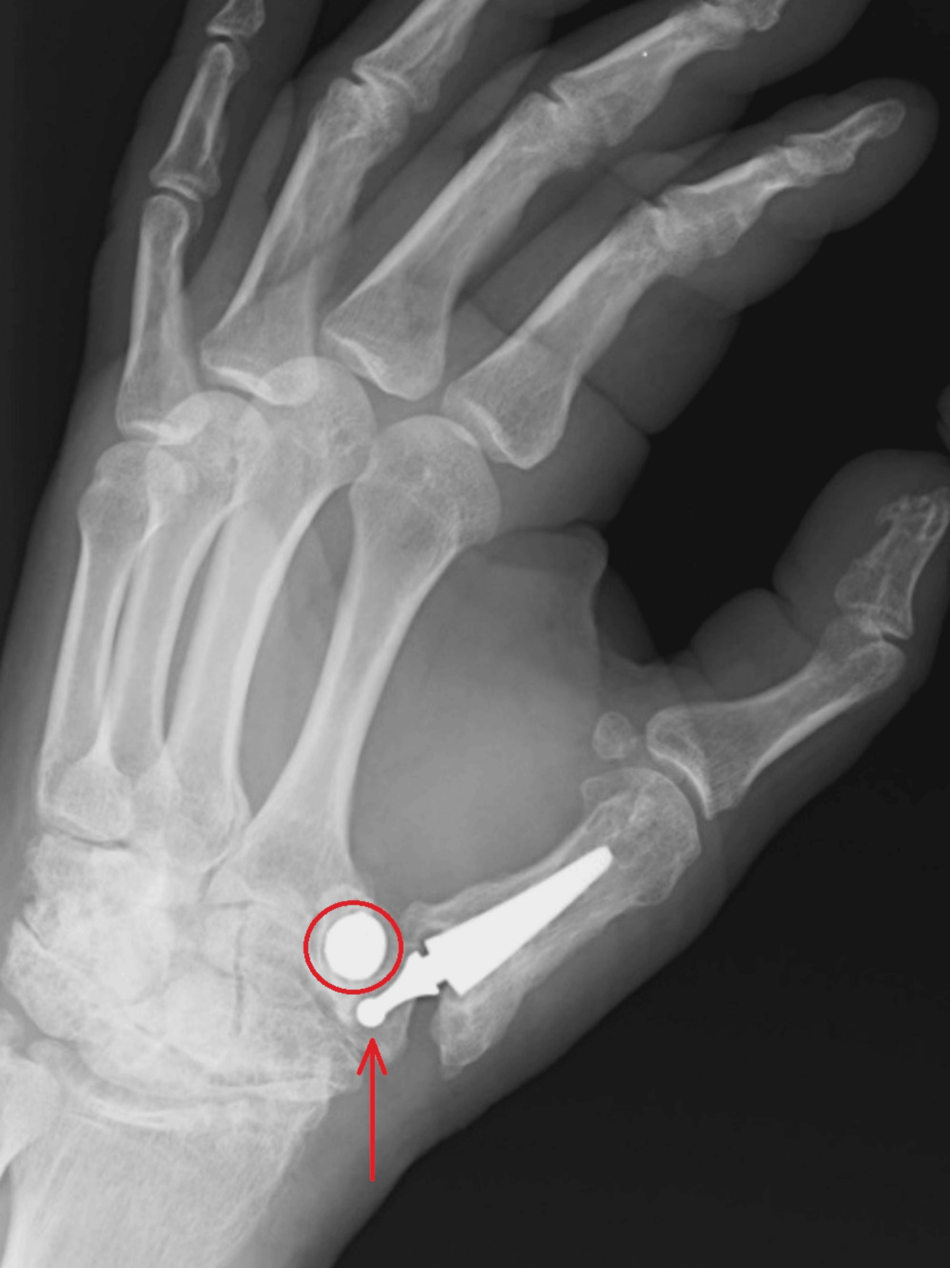
X-ray in Robert's projection obtained three years after surgery The figure shows dislocation of the implanted endoprosthesis elements. The red arrowhead marks the dislocated head of a double mobile CMC thumb prosthesis. The open circle marks the metal acetabular shell.

Three years after the surgery, as a result of an injury (falling down the stairs), the endoprosthesis dislocated, as shown in Figures 3, 4.

The patient initially downplayed the problem, and only after three months, when the swelling had subsided, did he notice a shortening of the operated thumb. He did not feel any pain in this area, only severe pain and limited mobility of the elbow joint of the operated limb. Based on the X-ray examination, post-traumatic changes in the elbow joint were excluded, but a dislocation of the Touch® prosthesis of the previously operated thumb was diagnosed. In this situation, a detailed assessment of the patient's clinical condition was made, which was based on the following research tools: the numeric rating scale (NRS) pain intensity scale, measurement of the range of motion in the sagital, frontal and opposition planes of the thumb, measurement of global grip strength, assessment of the functional state of the limb using the disabilities of the arm, shoulder, and hand (DASH) questionnaire. The examination showed that the patient's clinical condition practically did not deteriorate. The patient obtained identical results in the following parameters: resting and functional pain intensity, range of motion in all assessed planes, and in the assessment of the functional status in DASH. The only parameter whose result deteriorated was the value of global grip strength. A detailed comparison of the results is presented in Table 1. The patient himself assessed his level of fitness as satisfactory. Therefore, the entire team decided to try conservative treatment of the patient. This was dictated primarily by the lack of pain, lack of swelling, and fully satisfactory mobility of the thumb.

**Table 1:** Results of the assessed parameters in subsequent observation periods NRS rest – numerical rating scale – assessing the intensity of resting pain; NRS functional - numerical rating scale – assessing the intensity of functional pain; range of motion in the sagittal and frontal plane – assessed using a goniometer; opposition of the thumb according to the Kapanji scale (0 – 10); global grip strength, broken limb – assessed using the SAEHAN hydraulic dynamometer (SAEHAN Corporation, Gyeongsangnam-do, Republic of Korea); DASH - Disabilities of the Arm, Shoulder and Hand questionnaire assessing the functional status of the upper limb

Parameter	24 months (before dislocation)	36 months (after dislocation)
NRS rest	1	0
NRS functional	0	0
Range of motion in the sagittal plane	45	45
Range of motion in the frontal plane	40	40
Opposition of the thumb according to Kapanji	10 (10)	10 (10)
Global grip strength, broken limb	51 kg	43 kg
DASH	4,5 (100)	4,5 (100)

## Discussion

Total arthroplasty of the CMC thumb joint is effective in treating pain. Most authors state that about 80% of their patients feel no pain or observe a very large improvement. In addition, patients observe an improvement in range of motion. Lemoine et al. report that 91% of patients had a Kapanji score of 8 or more, and 65% had a score of 10. Unfortunately, like any surgical treatment, it always carries a risk of various complications. One of them is prosthesis dislocation. Lemoine et al. did not observe any case of such a complication in the studied group treated with the second-generation GUEPAR implant [3]. Gómez-Garrido et al. evaluated the long-term results of 137 patients (151 hands) with degenerative disease of the thumb CMC joint. In 5 cases (3.6%), traumatic dislocation of the prosthesis occurred within 5 years [4]. Froschauer et al. evaluated the implantation of 40 prostheses. In one patient, dislocation of the prosthesis was noted 7 days after surgery, caused by a too-short prosthesis neck [5]. Farkash et al. performed 381 implantations of CMC joint endoprostheses between 2015 and 2022. The authors found that major complications occurred in 6% and minor complications in 25.4% of patients. The vast majority of complications occurred within the first six months after surgery [6]. Dislocation rates reported in the literature are as high as 15% and may be the result of implant malposition, bone compression, soft tissue failure, postoperative rehabilitation, and wear. Lemoine et al. report that a higher risk of dislocation may be related to the type of prosthesis used and is particularly true for nonretentive implants with a wide range of motion. In addition, wear of the polyethylene cup may cause it to lose its retentiveness and increase the risk of dislocation [3]. The risk of dislocation in the dual mobility endoprosthesis model we used is also described as low. Despite this, this complication occurred in the described case, which was most likely caused by trauma (falling down stairs).

The established procedures suggest that in the case of an early diagnosed dislocation of the CMC thumb prosthesis, the patient qualifies for simple reposition of the dislocation, which is performed in the operating room. Additionally, under X-ray monitoring, the correctness of the implant placement and its stability and compatibility are assessed. In a situation where it is impossible to reposition the dislocated CMC joint prosthesis using the simple reposition method, its loosening is recognized, dislocation of one of the prosthesis elements is confirmed, or the presence of a too-short prosthesis neck is confirmed, the patient qualifies for surgical treatment. During open reduction, depending on the diagnosed problem, a simple prosthesis adjustment is performed, and loose elements or the neck are replaced. The procedure is different in the case of a persistent dislocation of the thumb CMC joint prosthesis. This is being discussed because the patient suffered an injury about six weeks earlier. If the patient reports after such a period of time, and the clinical examination indicates limited mobility and pain, he or she is immediately qualified for surgical treatment. However, in the case of a persistent dislocation without current pain and no limitation of joint mobility, it seems reasonable to refrain from surgical intervention. Such a patient also qualifies for further regular outpatient check-ups. This is the procedure suggested to our patient. The choice of this treatment method results from the fact that each revision procedure carries a higher risk of postoperative complications, which is confirmed by our experience and the observations of other authors. For example, Hansen et al. report that replacement of a CMC thumb prosthesis is a difficult operation with a steep learning curve [7].

## Conclusions

Dislocation of the thumb CMC joint prosthesis in a patient with a previous Rolando fracture did not affect the clinical condition of the hand. The patient obtained identical results in the following parameters: resting and functional pain intensity, range of motion in all assessed planes, and in the assessment of functional status using the DASH questionnaire. From the assessed parameters, only a slight decrease in the global grip strength of the hand was observed. The use of conservative treatment and observation allowed for the avoidance of a complicated revision surgery.
